# Uncommon Therapeutic Approaches for Patients with Recurrent Central Serous Chorioretinopathy: case report and literature review

**DOI:** 10.22336/rjo.2025.04

**Published:** 2025

**Authors:** Raluca Neacșa, Daniela Manasia, Mădălina-Elena Tobă

**Affiliations:** 1 Department of Medico-Surgical Disciplines, Ophthalmology, Titu Maiorescu University of Bucharest, Faculty of Medicine, Bucharest, Romania; 2 Department of Medico-Surgical Disciplines, General Surgery, Titu Maiorescu University of Bucharest, Faculty of Medicine, Bucharest, Romania; 3 Department of Ophthalmology, “Witting” Clinical Hospital, Bucharest, Romania

**Keywords:** central serous chorioretinopathy, Anti-VEGF injections, vision loss, BCVA = Best corrected visual acuity, CSCR = Central serous chorioretinopathy, IOP-OU = Both eyes intraocular pressure, OD = Right eye, OCT = Optical coherence tomography, OS = Left eye, PDT = Photodynamic therapy, RPE = Retinal pigment epithelium, VEGF = Vascular endothelial growth factor

## Abstract

**Objectives:**

The objective of anti-VEGF (Vascular Endothelial Growth Factor) injections in the treatment of central serous chorioretinopathy (CSCR) is to help reduce fluid accumulation beneath the retina, promote resolution of retinal edema, and potentially prevent complications like permanent vision loss.

The goal is to stabilize or improve vision by improving visual outcomes by reducing fluid and promoting retina reattachment.

**Case presentation:**

A 43-year-old Caucasian male presented with painless, decreased vision in the left eye. The diagnosis of central serous chorioretinopathy was made. After the anti-VEGF injections, the results were good, beyond expectations, and the OCT aspect was maintained at 6 months within normal limits of the foveal affected area without recording extra vision loss.

**Discussions:**

In CSCR, the retinal pigment epithelium (RPE) becomes compromised, causing fluid from the choroid to leak under the retina, leading to retinal or serous detachment of the macula. While the exact cause of CSCR is not fully understood, it is believed that the increased permeability of the choroidal vessels, often due to elevated VEGF levels, contributes to fluid leakage. The specific goals of using anti-VEGF injections in CSCR include reducing choroidal vascular leakage, promoting fluid reabsorption, and preventing recurrence or progression.

**Conclusion:**

Anti-VEGF agents like aflibercept and ranibizumab are commonly used in treating CSCR, though their effectiveness can vary, and treatment regimens depend on individual patient response. Typically, anti-VEGF injections are administered via intravitreal injection into the eye, with repeat injections, if necessary, based on monitoring the patient’s condition.

## Introduction

Albrecht von Graefe first described the disease as central recurrent syphilitic retinitis in 1866. Over the next almost hundred years, it was attributed to many names, such as idiopathic flat detachment of the macula, central angiospastic retinopathy, and central serous retinopathy, until 1967, when Gass described it as idiopathic central serous chorioretinopathy [1].

Central serous chorioretinopathy (CSCR) is an eye disease characterized by fluid accumulation beneath the retina, specifically in the macula area, which can lead to visual distortion and loss.

This condition is one of the more common conditions affecting the retina, particularly in young to middle-aged adults.

During diagnosis, patients might complain of central distortion or vision loss, micropsia, hypertonic/myopic shift, and rescued contrast sensitivity and color saturation [2].

Clinical diagnosis highlights a best corrected visual acuity (BCVA) varying from 20/20 to 20/200.1, potentially a minimal afferent pupillary defect, and decreased critical flicker-fusion thresholds, which quickly improve once CSCR is resolved [3].

Ophthalmoscopy discloses a serous macular detachment without hemorrhage, and OCT discloses an RPE detachment, macular RPE mottling being frequently associated with chronic CSCR [4].

The prognosis for acute CSCR is good, with recovery of visual acuity within three months. Unfortunately, the documented recurrence is approximately 50% in the first year [5]. Other chronic disease prognostic markers include the persistence of symptoms and sub-retinal fluid for more than six months and total foveal thickness correlated with BCVA [6].

## Case presentation

A clinical case of a patient with CSCR who has had multiple recurrences to classical treatments and low access to other therapies is presented. The patient was treated with anti-VEGF therapy to decrease vascular leakage and help fluid absorption [7].

A 43-year-old Caucasian male presented for an ophthalmologic consult with decreased vision in the left eye; the patient was referred to our clinic with a suspicion of left eye idiopathic central serous chorioretinopathy. His medical background showed no personal or family history of ocular pathology or any other remarkable comorbidities related to his symptoms.

**Fig. 1:**
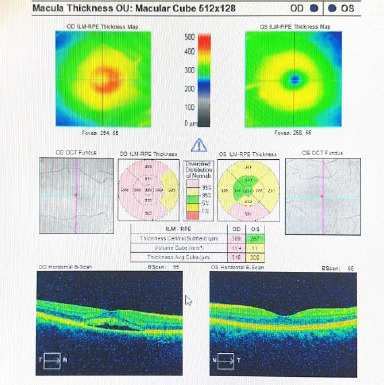
The OCT image of the central macula taken during first consult

During the first examination, a loss of visual acuity was found, with BCVA OD 20/20, BCVA OS 20/30, and IOP-OU=17 mmHg. Nothing abnormal was found after testing ocular motility, pupil, and confrontational fields. Slit lamp examination revealed a routine eye exam and dilated fundus exam revealed one large focal area of retinal hypo and hyperpigmentation in the left eye. Further investigation with an OCT of the macula head was performed, and some defects in the left eye with serous retinal detachment due to subretinal fluid were observed. The right eye examination revealed a normal foveal area with no remarkable aspects (**Fig. 1**).

Upon reviewing the clinical and paraclinical investigations and the patient’s medical history, he was diagnosed with central serous chorioretinopathy in the left eye.

The usual protocol for managing these cases consists of monitoring the patient status for 3-6 months, followed by oral treatments such as spironolactone. Still, because the patient was young and had significant vision loss, the decision was made that he should start treatment with focal laser photocoagulation to treat areas of active leakage, but not before performing fluorescein angiography on his left eye.

**Fig. 2:**
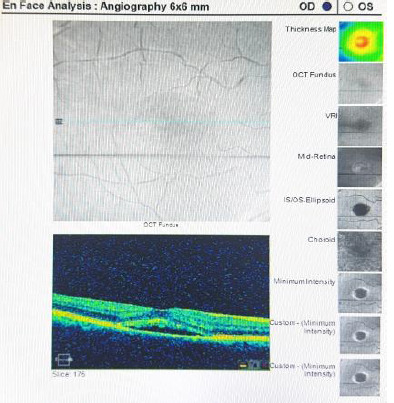
The OCT Angiography

Performing fluorescein angiography revealed early signs of hyperfluorescence, late pooling, and scattered staining with focal areas of atrophy (**Fig. 2**).

**Fig. 3:**
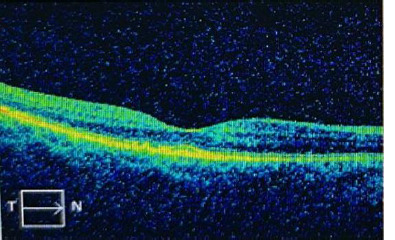
The OCT image of the macula after the focal laser photocoagulation treatment

After focal laser photocoagulation treatment, the evolution was excellent, with fluid reduction. Also, the tomographic appearance improved utterly, and the visual acuity recovered to 20/20 Schnelle (**Fig. 3**).

**Fig. 4:**
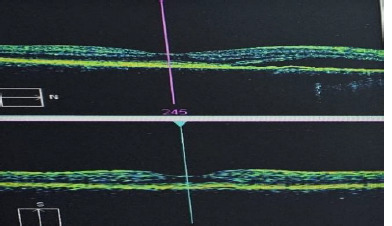
The OCT of the macula showing subretinal parafoveolar fluid

Two months after the focal laser photocoagulation treatment, the patient lost clarity and visual acuity again, and the OCT of the left eye's macula showed central subretinal fluid located para foveolar (**Fig. 4**).

Post-treatment, the results were unsatisfactory, and the evolution was unfavorable. As a consequence, anti-VEGF injection was considered in managing the disease.

The following treatment included one anti-VEGF injection in the left eye. The results after 1,3, and 6 months were good beyond expectations, and the OCT aspect was maintained at 6 months within normal limits of the foveal affected area without recording extra vision loss.

The prognosis was favorable without any other retinal lesions, and the patient was scheduled for OCT monitoring every six months.

## Discussions

Treatment options can vary based on the severity and duration of the condition. Central serous chorioretinopathy (CSCR) leads to visual distortion and impairment. While many cases resolve spontaneously within a few months, various treatment options exist for persistent or severe cases. The following is a detailed overview of treatment strategies and common approaches:

1. Observation: Careful monitoring without immediate treatment is recommended for many patients, especially those with mild symptoms or recent onset. Most cases resolve within 3 to 6 months [8].
2. Lifestyle Modifications: Patients are advised to manage or reduce stress, as psychological stress has been associated with CSCR. Avoiding corticosteroids and managing underlying health conditions (like hypertension) can be beneficial [9].
3. Oral Medications: Some medications, like mineralocorticoid receptor antagonists, may be prescribed to reduce fluid retention in specific cases. Drugs like spironolactone may be used to decrease fluid retention in chronic cases [10].
4. Photodynamic Therapy (PDT): This treatment uses a light-sensitive drug and a laser to target abnormal blood vessels that can contribute to fluid leakage. PDT involves the intravenous administration of a photosensitizing agent (like verteporfin) followed by laser activation. This treatment targets and reduces abnormal choroidal vascular permeability. Studies on the efficacy of this procedure show that PDT can lead to faster fluid resolution and improved visual outcomes, especially in chronic cases [11].
5. Laser Therapy: In some cases, thermal laser treatment can help seal leaks in the retinal layers. Focal laser photocoagulation can be used to treat areas of active leakage. However, it is less commonly used today due to the rise of PDT and anti-VEGF treatments. The final visual outcome following laser photocoagulation is not significantly improved [12].
6. Surgery: Surgical options are rarely considered for chronic or severe cases. In cases where conservative treatments fail, surgical interventions such as subretinal fluid drainage or other advanced techniques may be considered [13].
7. Anti-VEGF Injections: These medications can help reduce fluid accumulation by targeting the vascular endothelial growth factor, although its use in CSCR is less common than in other retinal diseases. Anti-VEGF agents (e.g., ranibizumab and aflibercept) can be considered for patients with persistent or recurrent CSCR, although their use is still being evaluated. The mechanism of these injections is to decrease vascular leakage and help in fluid absorption [14]. Anti-VEGF agents are commonly used in treating CSCR, though their effectiveness can vary, and treatment regimens depend on individual patient response. Typically, anti-VEGF injections are administered via intravitreal injection into the eye, with repeated injections, if necessary, based on monitoring the patient’s condition.

The specific goals of using anti-VEGF injections in CSCR include:

1. Reducing choroidal vascular leakage by inhibiting VEGF, which is responsible for increasing vascular permeability; anti-VEGF injections help reduce fluid leakage under the retina, improving retinal structure and function.
2. Promoting fluid reabsorption: Anti-VEGF therapy can help the retina reabsorb the fluid that has accumulated beneath the macula, decreasing retinal edema and improving vision.
3. Preventing recurrence or progression: In some cases, repeated anti-VEGF injections may be used to prevent the recurrence of fluid buildup or the development of chronic or more severe CSCR, especially in cases resistant to other treatments.
4. Improving visual outcomes: The goal is to stabilize or improve vision by reducing fluid and promoting retina reattachment. While not all patients experience dramatic vision improvement, many see stabilization or some vision recovery after treatment.

## Conclusion

Treatment for CSCR should be individualized based on the patient’s condition, duration of symptoms, and overall health. Regular follow-ups with an eye care professional are crucial for monitoring progress and adjusting treatment.

Although eye care professionals have not reached a consensus regarding the efficiency of anti-VEGF in managing CSCR, these therapies are considered an option for patients who respond poorly to other treatment options or have limited access to other therapies.
